# Accelerating Brain MR Imaging With Multisequence and Convolutional Neural Networks

**DOI:** 10.1002/brb3.70163

**Published:** 2024-11-17

**Authors:** Zhanhao Mo, He Sui, Zhongwen Lv, Xiaoqian Huang, Guobin Li, Dinggang Shen, Lin Liu, Shu Liao

**Affiliations:** ^1^ Department of Radiology China‐Japan Union Hospital of Jilin University Changchun China; ^2^ Shanghai United Imaging Intelligence Co. Ltd. Shanghai China; ^3^ Shanghai United Imaging Co. Ltd. Shanghai China

**Keywords:** accelerated imaging, convolutional neural networks, deep learning, magnetic resonance imaging

## Abstract

**Purpose:**

Magnetic resonance imaging (MRI) refers to one of the critical image modalities for diagnosis, whereas its long acquisition time limits its application. In this study, the aim was to investigate whether deep learning–based techniques are capable of using the common information in different MRI sequences to reduce the scan time of the most time‐consuming sequences while maintaining the image quality.

**Method:**

Fully sampled T1‐FLAIR, T2‐FLAIR, and T2WI brain MRI raw data originated from 217 patients and 105 healthy subjects. The T1‐FLAIR and T2‐FLAIR sequences were subsampled using Cartesian masks based on four different acceleration factors. The fully sampled T1/T2‐FLAIR images were predicted from undersampled T1/T2‐FLAIR images and T2WI images through deep learning–based reconstruction. They were qualitatively assessed by two senior radiologists in accordance with the diagnosis decision and a four‐point scale image quality score. Furthermore, the images were quantitatively assessed based on regional signal‐to‐noise ratios (SNRs) and contrast‐to‐noise ratios (CNRs). The chi‐square test was performed, where *p* < 0.05 indicated a difference with statistical significance.

**Results:**

The diagnosis decisions from two senior radiologists remained unchanged in accordance with the accelerated and fully sampled images. There were no significant differences in the regional SNRs and CNRs of most assessed regions (*p* > 0.05) between the accelerated and fully sampled images. Moreover, no significant difference was identified in the image quality assessed by two senior radiologists (*p* > 0.05).

**Conclusion:**

Deep learning–based image reconstruction is capable of significantly expediting the brain MR imaging process and producing acceptable image quality without affecting diagnosis decisions.

## Graphical abstract

Deep learning‐based rapid MR reconstruction was assessed on the T1‐FLAIR and T2‐FLAIR sequences, and its performance was compared with that of conventional reconstruction strategies. Both quantitative and qualitative analyses were performed on the deep learning‐based MR reconstruction results across various acceleration factors, revealing image quality comparable to that of fully sampled images. Images produced through deep learning‐based reconstruction exhibit reduced artifacts and enhanced soft tissue contrast compared to those generated by conventional reconstruction methods.

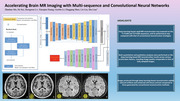

**Abbreviations**
CNR: contrast‐to‐noise ratio
CSF: cerebrospinal fluid
FLAIR: fluid‐attenuated inversion recovery
LSGAN: least squared generative adversarial network
PACS: picture archiving and communication system
ROI: region of interest
SNR: signal‐to‐noise ratio
T2WI: T2‐weighted image

## 1 Introduction

Magnetic resonance imaging (MRI) plays a crucial role in accurate disease diagnosis due to its unique advantages, such as being radiation‐free with superior soft tissue contrast and providing complementary anatomy information through different sequence designs. However, its slow acquisition speed leads to patient discomfort and low hospital throughput. Therefore, there is an urgent need for accelerating MRI scans that have garnered widespread attention over decades.

The most common way to expedite an MRI scan is to undersample the k‐space from which raw MRI signals are obtained (Twieg 1983). However, undersampling the k‐space will cause aliasing and artifacts in the resulting image since it violates the Nyquist–Shannon constraint. Several strategies have been proposed to address this problem. For instance, a possible way is to explicitly enforce data redundancy (e.g., parallel imaging; Zhu et al. 2022). Representative parallel imaging methods comprise SENSitivity Encoding (SENSE) (Kim et al. 2022), GeneRalized Autocalibrating Partial Parallel Acquisition (GRAPPA) (Lingala et al. 2017), and so forth. SENSE considers the spatial sensitivities of each coil, and the aliased images are unwrapped in the real space. Besides, GRAPPA accelerates the reconstruction in the complex frequency domain before the signal transforms into the image space. Compressed sensing refers to another stream of MRI fast reconstruction methods. It assumes that MRI data follow the sparse representation in several transform domains, and the image can be recovered through iterative optimization. The most used transform domain comprises the image domain and the wavelet domain. Furthermore, other sparsity constraints (e.g., the dictionary‐based sparsity and low‐rank sparsity) were adopted.

In recent years, deep‐learning techniques have made significant strides in image analysis and reconstruction (Anwar, Abrar, and Ullah 2023; Bin Tufail et al. 2021; Rasheed et al. 2024; Rasheed et al. 2023; Ullah et al. 2023a, 2023b, 2024, 2023c), particularly in fast MRI reconstruction. For example, the AUtomated TransfOrm by Manifold APproximation method, based on deep learning, directly reconstructs images from raw signals (Ahmed et al. 2021). The use of a deep cascade strategy has been employed for dynamic MRI reconstruction to enforce data consistency constraints in the frequency domain sequentially; this approach was subsequently extended to incorporate recurrent neural networks for more precise dynamic MRI reconstruction. In addition, the variational network has been proposed for rapid MRI reconstruction (Wang et al. 2022). Furthermore, generative adversarial networks (GANs) have been widely utilized for fast MRI reconstruction (Choi et al. 2022). GAN‐based frameworks rely on the evolution of both generator and discriminator networks to automatically enhance image contrast and appearance. Representative GAN‐based methods include de‐aliasing GAN and context‐aware GAN (Joshi et al. 2022; Yang et al. 2018).

Nevertheless, there remains uncertainty regarding the impact of deep learning–based rapid MRI reconstruction within clinical settings due to a predominant focus on publicly available data from healthy subjects with consistent anatomical features. This investigation evaluates both qualitatively and quantitatively how effective deep learning–based rapid MRI reconstruction techniques are for brain imaging. Our emphasis lies on expediting fluid‐attenuated inversion recovery (FLAIR) sequences, given their significance, despite being time‐consuming during routine brain MRIs. Furthermore, leveraging multisequence information has shown potential for enhancing deep learning–based reconstruction quality; thus, our study employs an enhanced network architecture. Specifically, we utilize a fully sampled T2‐weighted image (T2WI) sequence (a standard component of routine brain MRIs acquired more swiftly than FLAIR sequences) alongside a downsampled FLAIR sequence as inputs for prediction by our network.

In this study, we aim to investigate the potential of deep learning–based techniques in harnessing shared information across different MRI sequences from the same patient to significantly reduce scan time for the most time‐consuming sequences, while upholding image quality for accurate diagnosis. This study makes three primary contributions: (1) introducing a deep learning–based reconstruction technique designed to expedite the time‐consuming FLAIR sequence using downsampled FLAIR and fully sampled T2WI sequences, (2) evaluating the proposed method on both the healthy subjects and the clinical patients to assess its effectiveness and generalizability; (3) conducting comprehensive quantitative and qualitative assessments of the proposed method, including exploration of various acceleration factors.

## 2 Materials and Methods

### 2.1 Cohort

**FIGURE 1:**
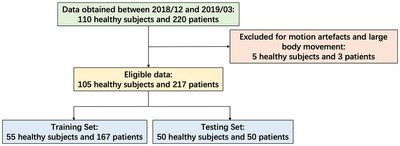
Flowchart of the data selection and exclusion criteria, and the separation of the training and testing set.

This study included 110 healthy adults and 220 patients (18 years or older) who underwent brain imaging between December 8, 2018 and March 11, 2019. The raw MRI data for both groups were stored in a workstation for over three months. Experiments were conducted using the T2WI, T1‐FLAIR, and T2‐FLAIR sequences. Five healthy subjects and three patients were excluded due to motion artifacts and whole‐body movement, respectively. Therefore, the final analysis included 105 healthy subjects and 217 patients (Figure 1). The patient pathologies are listed in Table 1; note that multiple pathologies may exist in a single patient. Subsampled T1‐FLAIR/T2‐FLAIR images were generated using Cartesian undersampling masks with acceleration factors of 3.0, 3.5, 4.0, and 4.5.

**TABLE 1:** The disease distribution of recruited patients.

Diagnoses	Number	Gender		Age (years, mean ± SD)
		Male	Female	
Encephalomalacia	9	4	5	63.33 ± 8.37
Chronic cerebral hemorrhage	9	6	3	60.54 ± 5.19
Massive Infarction	13	8	5	64.29 ± 8.86
Lacunar infarction	148	73	75	62.32 ± 13.61
Demyelination	95	40	55	62.49 ± 15.71
Arachnoid cyst	5	2	3	60.14 ± 14.95

### 2.2 MRI Protocol

All patients were examined by a 3T scanner (uMR780, United Imaging Healthcare, Shanghai, China). It includes three brain imaging protocols using a 24‐channel head coil (HNC‐24). A two‐dimensional T2‐weighted sequence with the following parameters: TR: 4220 ms, TE: 104.8 ms, FA: 125°, FOV: 230 mm × 200 mm, acquired voxel size: 0.60 mm × 0.60 mm × 5.00 mm, and plane: axial. A two‐dimensional T1‐weighted FLAIR sequence with the following parameters: TR: 2015 ms, TE: 12.6 ms, TI: 860 ms, FA: 135°, FOV: 230 mm × 200 mm, acquired voxel size: 0.73 mm × 0.65 mm × 5.00 mm, and plane: axial. A two‐dimensional T2‐weighted FLAIR sequence with the following parameters: TR: 8000 ms, TE: 105 ms, TI: 2465, FA: 150°, FOV: 230 mm × 200 mm, acquired voxel size: 0.95 mm × 0.76 mm × 5.00 mm, and plane: axial.

### 2.3 Deep Learning–Based Reconstructions

In this study, image data were processed on a computer equipped with Ubuntu 16.04 as the operating system, utilizing the PyTorch platform for implementing the deep‐learning reconstruction technique, with CUDA 8.0 and cuDNN 7.0. The system was architecturally robust, featuring an Intel Xeon E5‐2620 v4 processor, clocked at 2.10 GHz, accompanied by a storage capacity of 4 TB, 128 GB of RAM. It was further empowered by 4 Nvidia TITAN XP GPUs, each with 12 GB, to accelerate computations. The training time was around 4 days for the proposed method.

**FIGURE 2:**
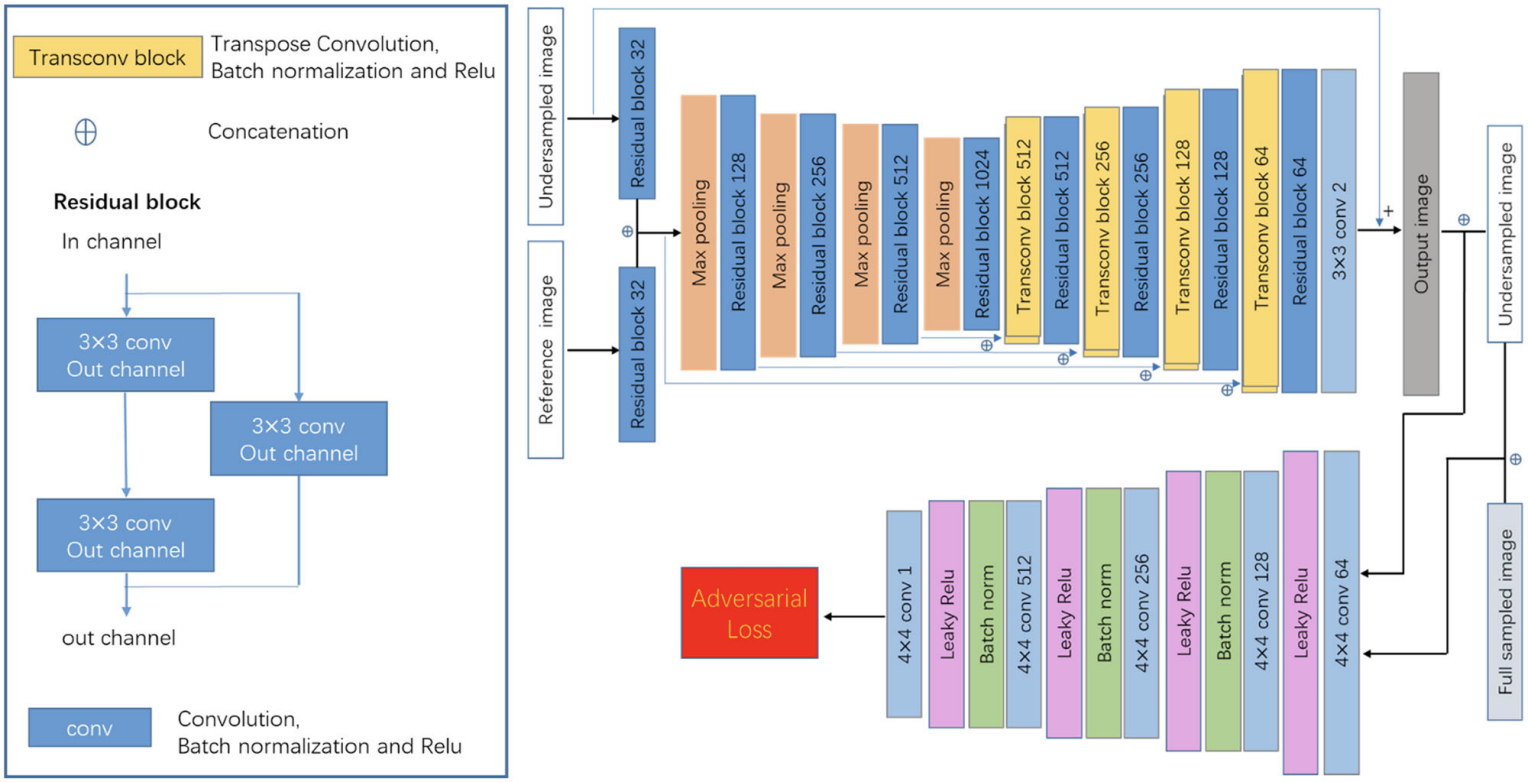
Network structure of the deep learning–based fast MR reconstruction framework used in this study.

We expanded upon the network architecture designed by Xiang et al. (2018), implementing a fully convolutional neural network that accepts dual inputs: the undersampled sequence targeted for reconstruction and a fully sampled reference. Specifically, the undersampled T1‐FLAIR/T2‐FLAIR sequence paired with the fully sampled T2WI sequence was fed into the network. Network input consisted of the real and imaginary components of these sequences following inverse Fourier transformation, and the goal was to output the reconstructed FLAIR image's real and imaginary parts. Both sequences first encountered residual blocks with 32 channels for initial feature extraction, with consequent concatenation to integrate reference sequence information into the downsampling sequence's reconstruction process. Following concatenation, a U‐net‐style architecture, influenced by the work of Ronneberger, Fischer, and Brox (2015), was employed for multiresolution information extraction, utilizing residual blocks together with standard convolutions. This structure incorporated four downsampling stages with max pooling and residual blocks, starting with 128 channels that doubled after each downsampling. Each residual block consisted of a 3 × 3 convolution layer, batch normalization, and ReLu activation. The network training was implemented in the PyTorch DL framework, using the ADAM optimizer with an initial rate of 0.0001, betas of (0.9, 0.999), and the learning rate decayed by half every 100 epochs. Conversely, the upsampling path, also with four stages, featured deconvolution and residual blocks, halving the channel count after each upsampling. To accelerate learning, a long skip connection facilitated the computation of residuals between fully sampled and undersampled images. Each sequence during scans contained multiple channels, processed sequentially by the network. To enhance reconstruction quality, the least squared generative adversarial network (LSGAN) training strategy was adopted (Ren et al. 2018). Figure 2 outlines these advancements. Importantly, the framework is adaptable to scenarios with a single available sequence, limiting inputs to just the undersampled sequence without altering the structure except for input adjustment. Nonetheless, when a fully sampled reference sequence is accessible alongside the undersampled one (i.e., typical in clinical settings with multiple scans), the methodology generally achieves superior reconstruction quality, especially under high acceleration factors, due to the valuable anatomical cues offered by the additional fully sampled data.

Out of the 105 healthy subjects and 217 patients, 50 random healthy subjects and 50 random patients were selected for testing. The remaining 222 subjects (55 healthy subjects and 167 patients) were randomly divided into a training set (consisting of 80% of the data, with 44 healthy subjects and 133 patients) and a validation set (20% of the data, with 11 healthy subjects and 34 patients). The training set was utilized to train the network, while the validation set was used to optimize model parameters. Four different acceleration factors (3.0×, 3.5×, 4.0×, and 4.5×) were considered to evaluate the proposed fast MR imaging method. The average inference speed to reconstruct one image was 0.4 s for our method.

### 2.4 Statistical Analysis and Assessment

#### 2.4.1 Scan and Reconstruction Time Comparison

First, the scan and reconstruction time was assessed using the conventional strategy and the proposed method. The GRAPPA scan and reconstruction strategy is adopted in the conventional strategy, which is one of the most used scan and reconstruction methods. The average total time for the conventional strategy (i.e., the scan and reconstruction time) corresponding to the T1‐FLAIR and T2‐FLAIR sequences reaches 124.2 and 131.4 s, respectively. The average total time required for the proposed method based on the 4.5× acceleration factor reaches 38.5 s (with a scan time of 26.2 s and a reconstruction time of 12.3 s) and 40.5 s (with a scan time of 27.8 s and a reconstruction time of 12.7 s), respectively, significantly shorter than average total time required by the conventional strategy. The reconstruction computer has an Intel Xeon CPU E5‐2620 v4 2.10‐GHz processor, 4 TB of hard disk, 128 GB of RAM, and 4 Nvidia TITAN XP x12 GB GPUs.

#### 2.4.2 Diagnosis Consistency Analysis

Two senior radiologists conducted a blinded review of fully sampled T1‐FLAIR/T2‐FLAIR images and deep learning–reconstructed images from healthy subjects or patients to make diagnostic assessments. The consistency between the diagnostic assessments based on the fully sampled and deep learning–reconstructed images was compared using Cohen's kappa correlation coefficient to examine the interobserver agreement in qualitative, in vivo assessment of pathologic indexes and visibility of anatomical structures.

#### 2.4.3 Quantitative Analysis

Regional quantitative image quality analysis was performed using the picture archiving and communication system workstation at our institution. Radiologists with 5 years of subspecialty experience drew regions of interest (ROIs) using the Horos software (https://horosproject.org/) on representative axial slices of conventional and accelerated images with acceleration factors of 3.0, 3.5, 4.0, and 4.5. The undersampling masks for different acceleration factors were generated by initially including a certain number of adjacent lowest frequency k‐space lines to ensure a fully sampled k‐space region, followed by uniform random inclusion of the remaining k‐space lines based on a preset probability that achieves the desired acceleration factor on average. The omission of k‐space lines was limited to the phase encoding direction to simulate physically achievable accelerations for 2D sequences. ROIs were delineated for grossly normal cerebrospinal fluid (CSF), white matter, gray matter, fat, muscle, and pathology, as well as surrounding pathology areas in consistent anatomical locations and sizes between conventional brain MRI and deep learning–reconstructed sequences presented side‐by‐side in the same patient‐involving areas; each ROI covered more than half the area of its corresponding lesion. Subsequently, signal‐to‐noise ratios (SNRs) and contrast‐to‐noise ratios (CNRs) were calculated from these ROIs. The SNRs for white matter, grey matter, fat tissue, rectus extraocular muscle tissue, and pathologic tissues, as well as their surrounding regions, were calculated by dividing the average signal intensity value within each ROI placed on the respective tissues (SI_tissue_) by their standard deviation values inside each ROI (SD_tissue_). Standard deviation values from within tissues were used instead of background due to nonuniformity across regions in an accelerated sparse image. $\mathrm{SNR}=SI_{\mathrm{tissue}}/SD_{\mathrm{tissue}}$ where SD_tissue_ denotes the average signal intensity value of ROI placed on the corresponding tissue and SD_tissue_ denotes the standard deviation of the corresponding tissue inside the ROI. The CSF, white matter, gray matter, fat, rectus extraocular muscle, pathology, and pathology surrounding CNRs were calculated by the following equation:

$$\mathrm{CNR}\left(\mathrm{tissu}e_{1},\;\mathrm{tissu}e_{2}\right)=\frac{\left|SI_{\mathrm{tissu}e_{1}}-SI_{\mathrm{tissu}e_{2}}\right|}{\sqrt{S{D_{\mathrm{tissu}e_{1}}}^{2}+S{D_{\mathrm{tissu}e_{2}}}^{2}}}$$

where SD_tissue1_ and SD_tissue2_ denote the average signal intensity value of ROI placed on two different tissues, while SD_tissue1_ and SD_tissue2_ denote the standard deviation of the two tissues inside their corresponding ROI.

Quantitative evaluation was performed by means of the chi‐square test.

#### 2.4.4 Qualitative Analysis

The axial accelerated and fully sampled T1WI/T2WI images were independently evaluated by two board‐certified neuroradiologists with 6 years of MR experience, specializing in neurological and oncological imaging. The readers were blinded to all identifying information about sequence type. Each reader independently assessed the following six features for the respective sequence:

1. Overall image quality
2. Gray–white matter delineation
3. Lesion delineation
4. Overall diagnostic usefulness
5. Contrast fat and rectus extraocular muscle
6. Presence and severity of susceptibility artifacts

Features 1–5 were rated on a 5‐point scale as follows: *excellent* = 4, *good* = 3, *fair* = 2, *poor* = 1, and *nondiagnostic* = 0. Feature 6 was evaluated on a 3‐point scale: *artifacts present and severe enough to impair diagnostic confidence* = 2, *artifacts present but not impairing diagnostic confidence* = 1, and *artifacts not present* = 0. The mean scores from both raters served as the representative value. The statistical significance of the qualitative data was determined using the Kruskal–Wallis nonparametric test.

#### 2.4.5 Statistical Analysis

Statistical analysis was performed using SPSS (IBM SPSS Statistics 23.0, Armonk, NY). The inter‐reader agreement for the qualitative scores was evaluated using Cohen's weighted kappa statistics. Interobserver agreement was considered less than chance when *k* < 0, slight when 0.01 < *k* < 0.20, fair when 0.21 < *k* < 0.40, moderate when 0.41 < *k* < 0.60, substantial when 0.61 < *k* < 0.80, and almost perfect when 0.81 < *k* < 0.99. Data regarding contrast ratio measurements are presented as mean scores with standard deviations. The statistical significance of contrast ratio measurements and qualitative ratings was determined through analysis of variance and the Kruskal–Wallis nonparametric test. *p* values less than 0.05 were deemed statistically significant.

## 3 Results

### 3.1 Diagnosis Consistency Results

**FIGURE 3:**
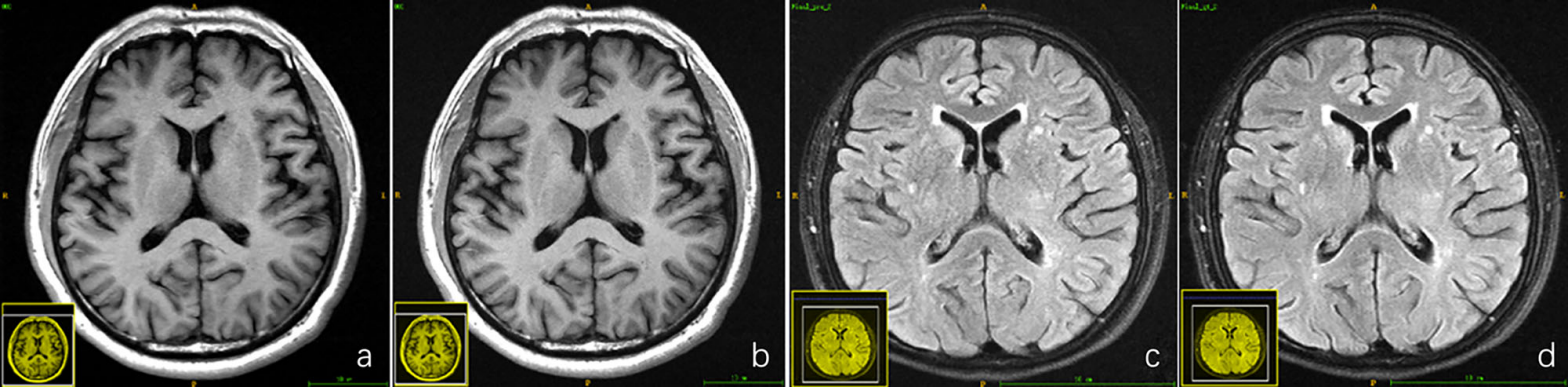
(a) Deep learning–based reconstruction T1‐FLAIR image from 4.5× acceleration factor, (b) the corresponding full sampled T1‐FLAIR image, (c) deep learning–based reconstruction T2‐FLAIR image with 4.5× acceleration factor, and (d) the corresponding full sampled T2‐FLAIR image. Both radiologists made the same diagnosis decision to the deep learning–reconstructed and full sampled T1‐FLAIR image as normal subject, and the deep learning–reconstructed and full sampled T2‐FLAIR image has bilateral basal ganglia.

For both the 50 healthy subjects and the 50 patients, both senior radiologists arrived at the same diagnostic conclusion based on the blinded reading experiment for the fully sampled T1‐FLAIR/T2‐FLAIR data and the deep learning–reconstructed T1/T2‐FLAIR data across all acceleration factors. Consequently, the robustness of the deep learning–based reconstruction algorithm was validated. The fully sampled T1‐FLAIR/T2‐FLAIR images and their deep learning–reconstructed counterparts are depicted in Figure 3, along with the diagnostic decisions made by the two radiologists.

### 3.2 Quantitative Results

The results of the quantitative analysis obtained from all the healthy subjects and clinical patients are listed in Tables 2 and 3. Bold indicates no significant difference in data.

**TABLE 2:** SNR comparison between the fully sampled T1/T2‐FLAIR and deep learning–based reconstruction results with respect to different acceleration factors for the volunteer and clinical group.

SNR	Full	DL 3.0×	DL 3.5×	DL 4.0×	DL 4.5×	*p*
**T1‐FLAIR volunteer**						
White matter	24.73 ± 4.7	31.72 ± 5.51	36.81 ± 7.56	34.56 ± 7.55	30.92 ± 7.33	< 0.01
Gray matter	12.91 ± 3.68	12.93 ± 3.97	13.82 ± 5.10	13.28 ± 4.12	11.37 ± 3.00	**0.510**
CSF	6.32 ± 0.84	3.68 ± 0.69	3.55 ± 0.70	3.59 ± 0.64	3.31 ± 0.74	< 0.01
Fat	12.08 ± 2.22	14.91 ± 3.55	15.42 ± 4.37	17.22 ± 4.70	16.01 ± 4.36	< 0.05
Muscle	8.08 ± 3.30	9.39 ± 3.93	9.91 ± 4.21	10.01 ± 4.06	10.71 ± 4.71	< 0.01
**T1‐FLAIR clinical**						
White matter	28.04 ± 4.94	34.63 ± 8.03	38.80 ± 7.58	36.30 ± 7.25	31.32 ± 6.25	< 0.01
Gray matter	14.88 ± 3.58	13.49 ± 3.62	16.08 ± 7.29	14.48 ± 4.79	13.45 ± 3.74	< 0.05
CSF	7.34 ± 0.77	4.28 ± 0.90	4.32 ± 0.76	4.03 ± 0.72	3.89 ± 0.64	< 0.01
Fat	14.76 ± 3.88	16.63 ± 4.49	20.81 ± 6.27	18.20 ± 5.28	17.77 ± 4.44	< 0.05
Muscle	16.35 ± 6.72	19.52 ± 9.07	22.09 ± 10.46	22.29 ± 10.6	21.00 ± 9.15	< 0.01
Pathology	15.37 ± 11.77	15.74 ± 13.20	17.93 ± 13.32	17.05 ± 14.2	16.05 ± 10.5	**0.854**
Pathology surrounding	25.02 ± 9.84	26.03 ± 7.95	35.26 ± 12.44	30.60 ± 14.27	26.97 ± 8.45	< 0.01
**T2‐FLAIR volunteer**						
White matter	22.08 ± 4.01	22.83 ± 5.14	22.12 ± 4.48	23.05 ± 4.44	22.84 ± 4.73	**0.745**
Gray matter	23.37 ± 6.54	23.59 ± 6.02	22.77 ± 6.27	22.90 ± 5.57	21.91 ± 5.02	**0.679**
CSF	5.75 ± 1.55	4.77 ± 0.89	4.70 ± 0.96	4.74 ± 0.91	4.64 ± 1.02	< 0.01
Fat	9.38 ± 2.14	9.62 ± 2.47	9.76 ± 2.40	9.46 ± 2.35	8.93 ± 2.33	**0.884**
Muscle	5.48 ± 1.46	5.41 ± 1.48	5.46 ± 1.75	5.50 ± 1.93	5.73 ± 1.32	**0.466**
**T2‐FLAIR clinical**						
White matter	19.55 ± 2.57	16.89 ± 1.97	17.90 ± 2.41	17.69 ± 2.64	16.03 ± 2.12	< 0.01
Gray matter	20.53 ± 3.06	17.68 ± 3.15	19.07 ± 3.35	18.39 ± 3.74	16.13 ± 3.05	< 0.01
CSF	6.18 ± 1.12	5.60 ± 0.73	5.56 ± 0.71	5.43 ± 0.70	5.17 ± 0.62	< 0.01
Fat	7.61 ± 1.22	7.83 ± 1.57	8.04 ± 1.47	7.59 ± 1.25	7.32 ± 1.30	**0.637**
Muscle	7.36 ± 1.79	7.60 ± 1.77	7.55 ± 1.90	7.23 ± 1.70	7.12 ± 1.65	**0.104**
Pathology	11.89 ± 6.78	11.19 ± 5.23	11.36 ± 5.34	11.44 ± 4.71	11.02 ± 4.97	**0.950**
Pathology surrounding	19.26 ± 3.85	16.97 ± 3.13	18.46 ± 3.57	17.82 ± 3.35	15.56 ± 2.53	< 0.01

*Note*: Bold indicates no significant difference data.

Abbreviations: 3.0× = downsampling factor of 3.0, 3.5× = downsampling factor of 3.5, 4.0× = downsampling factor of 4.0, 4.5× = downsampling factor of 4.5, CSF = cerebrospinal fluid, DL = deep learning–based reconstruction.

**TABLE 3:** CNR comparison between the fully sampled T1/T2‐FLAIR and deep learning–based reconstruction results with respect to different acceleration factors for the volunteer and clinical group.

CNR	Full	DL 3.0×	DL 3.5×	DL 4.0×	DL 4.5×	*p*
**T1‐FLAIR volunteer**						
White/gray matter	9.05 ± 2.20	10.36 ± 2.64	11.62 ± 3.66	10.77 ± 2.78	9.55 ± 2.10	< 0.01
White matter/CSF	18.43 ± 3.95	22.55 ± 3.87	25.75 ± 5.68	24.97 ± 5.33	21.74 ± 5.65	< 0.01
Gray matter/CSF	7.74 ± 2.4	8.55 ± 2.30	9.16 ± 2.87	9.15 ± 2.58	7.63 ± 1.92	< 0.01
Fat/muscle	4.23 ± 2.23	4.99 ± 2.68	5.38 ± 2.95	5.34 ± 2.84	5.65 ± 3.29	**0.117**
**T1‐FLAIR clinical**						
White/gray matter	7.99 ± 1.50	8.47 ± 1.99	9.63 ± 3.31	8.89 ± 2.40	8.54 ± 3.27	< 0.05
White matter/CSF	20.34 ± 3.37	23.65 ± 4.76	27.22 ± 5.08	25.23 ± 5.11	22.52 ± 3.54	< 0.01
CSF/pathology	9.11 ± 5.57	9.73 ± 5.47	12.02 ± 7.3	11.16 ± 7.23	10.57 ± 5.99	< 0.05
Pathology/surrounding	8.16 ± 6.57	8.44 ± 8.77	8.23 ± 6.85	7.74 ± 6.91	7.53 ± 6.98	< 0.05
Gray matter/CSF	9.37 ± 2.09	9.39 ± 2.38	10.93 ± 3.69	10.11 ± 2.64	9.44 ± 2.41	**0.174**
Fat/muscle	9.87 ± 4.37	11.95 ± 5.89	13.25 ± 6.47	13.23 ± 6.40	12.58 ± 5.70	**0.971**
**T2‐FLAIR volunteer**						
White/gray matter	1.74 ± 1.15	1.82 ± 1.28	1.75 ± 1.30	1.76 ± 1.20	1.65 ± 1.17	**0.974**
White matter/CSF	5.72 ± 3.99	5.96 ± 1.38	5.72 ± 1.42	5.93 ± 1.34	5.67 ± 1.30	**0.944**
Gray matter/CSF	6.33 ± 4.89	6.54 ± 1.38	6.25 ± 1.51	6.50 ± 1.44	6.19 ± 1.38	**0.952**
Fat/muscle	2.71 ± 1.12	2.95 ± 1.11	2.97 ± 1.19	2.91 ± 1.04	2.87 ± 0.97	**0.772**
**T2‐FLAIR clinical**						
White/gray matter	1.94 ± 1.05	1.73 ± 0.93	1.87 ± 1.01	1.85 ± 1.03	1.61 ± 0.91	**0.474**
White matter/CSF	12.03 ± 1.98	10.63 ± 1.40	11.40 ± 1.56	11.07 ± 1.61	10.02 ± 1.40	< 0.01
CSF/pathology	9.19 ± 4.76	8.59 ± 3.71	8.91 ± 3.98	8.91 ± 3.61	8.46 ± 3.64	< 0.01
Pathology/surrounding	3.36 ± 2.82	2.93 ± 2.16	3.01 ± 2.24	2.97 ± 2.05	2.83 ± 1.97	**0.999**
Gray matter/CSF	13.46 ± 1.86	11.81 ± 1.64	12.83 ± 1.76	12.30 ± 1.98	10.80 ± 1.55	**0.905**
Fat/muscle	1.18 ± 1.06	1.18 ± 1.08	1.21 ± 1.17	1.19 ± 1.06	1.15 ± 1.14	**0.811**

*Note*: Bold indicates no significant difference data.

Abbreviations: 3.0× = downsampling factor of 3.0, 3.5× = downsampling factor of 3.5, 4.0× = downsampling factor of 4.0, 4.5× = downsampling factor of 4.5, DL = deep learning–based reconstruction.

**FIGURE 4:**
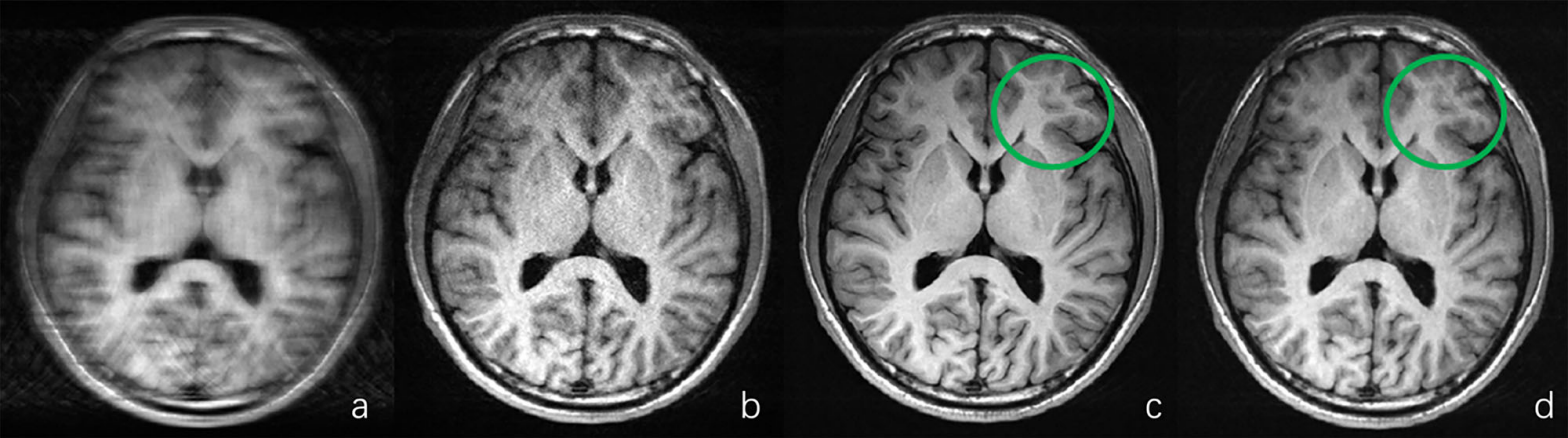
Typical example of (a) zero‐filled reconstruction, (b) parallel imaging reconstruction, and (c) deep learning–based reconstruction result of T1‐FLAIR image with 4.5× acceleration factor. (d) This shows the full sampled reconstruction result. Note that Gibbs artifacts are suppressed by the deep learning–based reconstruction result compared to the full sampled image, and significant differences are highlighted by the green circle.

**FIGURE 5:**
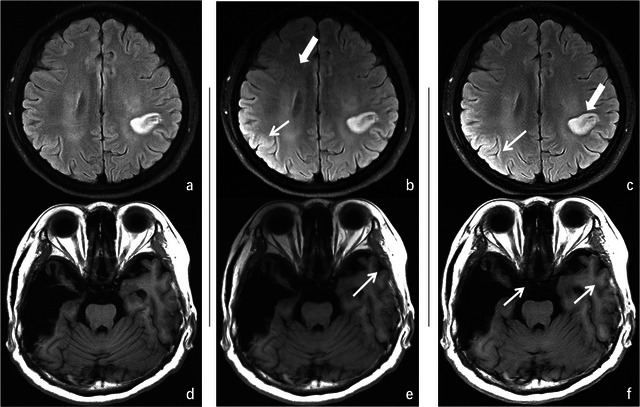
(a) Deep learning–based reconstruction of T2‐FLAIR images with a 4.5× acceleration factor; (b) VARNET reconstructed T2‐FLAIR image, showing areas of apparent signal enhancement in the cerebral cortex (thin arrows), while artifact correction remains suboptimal (thick arrows); (c) CycleGAN‐based network reconstructed T2‐FLAIR image, exhibiting distortion in both signal and extent of the lesion; (d) deep learning–based reconstruction of T1‐FLAIR images with a 4.5× acceleration factor; (e) VARNET reconstructed T1‐FLAIR image; (f) CycleGAN‐based network reconstructed T1‐FLAIR image, the artifact correction in the last two images is suboptimal, and their overall sharpness remains inadequate.

Based on the quantitative analysis results, it is evident that the deep learning–reconstructed T1‐FLAIR images exhibit statistically similar or even higher SNR and CNR values than the fully sampled images for most assessment metrics (e.g., white matter, fat, and muscle). This can be attributed to the effective suppression of Gibbs artifacts and enhancement of anatomical sharpness achieved through deep learning–based reconstruction. Figure 4 illustrates a representative example of T1‐FLAIR image reconstructions using zero‐filled, parallel imaging, and deep learning–based methods with an acceleration factor of 4.5×. In addition, the fully sampled image is included for comparison purposes. As depicted in the figure, the deep learning–based reconstruction clearly outperforms both zero‐filled and parallel imaging reconstructions. Furthermore, it effectively suppresses Gibbs artifacts present in the fully sampled image, particularly in areas highlighted by green circles. This observation aligns with quantitative assessment results presented in Tables 2 and 3, where certain metrics demonstrate superior performance of deep learning–based reconstruction compared to fully sampled images (e.g., SNR for white matter, fat, and muscle; CNR for white/gray matter and white matter/CSF). The SNR for CSF was lower after deep learning–based reconstruction compared to that of the fully sampled T1‐FLAIR image, as indicated in Table 2, due to its weak signal strength in FLAIR sequences, which is further distorted during the downsampling acceleration process. However, as shown in Figure 5, despite this limitation, the quality of the CSF region after deep learning–based reconstruction still surpasses conventional methods guiding radiologists toward accurate diagnostic decisions. For T2‐FLAIR reconstruction results, Table 2 indicates that the SNR of white matter and gray matter of the deep learning–based reconstructed results decreased with different acceleration factors. The possible reason for this is that T2‐FLAIR was more sensitive to lesions, and the signal strength of structural anatomies became weaker and more vulnerable during the acceleration process. Tables 2 and 3 also indicate that the differences of SNR for pathological regions and CNR between the pathological regions and surrounding structures compared with the fully sampled images were smaller, partially indicating the effectiveness of the deep learning–based reconstruction with respect to the lesion regions in T2‐FLAIR sequences. It was consistent with the results shown in the diagnosis consistency study in Section 3.1 that the reconstructed images from different acceleration factors led to the same diagnosis decision as the fully sampled images made by the two senior radiologists.

As shown in Tables 2 and 3, the SNR for certain anatomical structures decreases following deep learning–based reconstruction, falling below that of fully sampled sequences. This is primarily attributed to the heightened susceptibility of anatomies such as CSF, which exhibit weak signal strength in FLAIR sequences, to distortion and artifacts resulting from the downsampling process during acceleration. Furthermore, a qualitative analysis was performed to assess interobserver agreement between two board‐certified neuroradiologists, aiming to investigate the potential loss of image signal impacting diagnostic decisions. The qualitative findings are detailed in the subsequent section.

### 3.3 Qualitative Results

Table 4 lists the results of the subjective image quality analysis obtained with interobserver agreement from all the healthy subjects and patients. The diagnostic interobserver agreement was all above the moderate level (i.e., *k* > 0.41) between the conventional and deep learning–based reconstruction results of different acceleration factors. In this study, overall image quality, motion artifacts, other artifacts, and image pulsation artifacts were assessed.

**TABLE 4:** Subjective image quality evaluation and comparison between the conventional and deep learning–based reconstruction results with respect to different acceleration factors.

	Full	DL 3.0×	DL 3.5×	DL 4.0×	DL 4.5×	*P*
**T1‐FLAIR clinical**						
Overall image quality	3.42 ± 0.50	3.34 ± 0.48	3.52 ± 0.51	3.58 ± 0.50	3.44 ± 0.50	0.14
Gray–white matter delineation	3.40 ± 0.50	3.36 ± 0.49	3.50 ± 0.51	3.46 ± 0.50	3.46 ± 0.50	0.65
Lesion delineation	3.52 ± 0.51	3.38 ± 0.49	3.44 ± 0.50	3.46 ± 0.50	3.42 ± 0.50	0.71
Overall diagnostic usefulness	3.44 ± 0.50	3.38 ± 0.49	3.38 ± 0.49	3.48 ± 0.51	3.34 ± 0.48	0.64
Contrast fat and rectus extraocular muscle	3.46 ± 0.50	3.40 ± 0.50	3.38 ± ‐0.49	3.50 ± 0.51	3.52 ± 0.51	0.56
Presence and severity of susceptibility artifacts	0.06 ± 0.24	0.04 ± 0.20	0.04 ± 0.20	0.04 ± 0.20	0.04 ± 0.20	0.99
**T2‐FLAIR clinical**						
Overall image quality	3.54 ± 0.50	3.54 ± 0.50	3.58 ± 0.50	3.50 ± 0.51	3.56 ± 0.50	0.95
Gray–white matter delineation	3.54 ± 0.50	3.56 ± 0.50	3.56 ± 0.50	3.50 ± 0.51	3.50 ± 0.51	0.95
Lesion delineation	3.52 ± 0.51	3.50 ± 0.51	3.54 ± 0.50	3.50 ± 0.51	3.48 ± 0.51	0.98
Overall diagnostic usefulness	3.48 ± 0.51	3.48 ± 0.51	3.54 ± 0.50	3.50 ± 0.51	3.50 ± 0.51	0.98
Contrast fat and rectus extraocular muscle	3.52 ± 0.51	3.50 ± 0.51	3.46 ± 0.50	3.52 ± 0.51	3.54 ± 0.50	0.95
Presence and severity of susceptibility artifacts	0.04 ± 0.20	0.06 ± 0.24	0.06 ± 0.24	0.04 ± 0.20	0.04 ± 0.20	0.97
**T1‐FLAIR volunteer**						
Overall image quality	3.50 ± 0.51	3.50 ± 0.51	3.56 ± 0.50	3.48 ± 0.51	3.54 ± 0.50	0.93
Gray–white matter delineation	3.54 ± 0.50	3.50 ± 0.51	3.50 ± 0.51	3.48 ± 0.51	3.54 ± 0.50	0.97
Overall diagnostic usefulness	3.52 ± 0.51	3.50 ± 0.51	3.52 ± 0.51	3.46 ± 0.50	3.52 ± 0.51	0.97
Contrast fat and rectus extraocular muscle	3.54 ± 0.50	3.48 ± 0.51	3.52 ± 0.51	3.48 ± 0.51	3.52 ± 0.51	0.97
Presence and severity of susceptibility artifacts	0.04 ± 0.20	0.06 ± 0.24	0.04 ± 0.20	0.06 ± 0.24	0.06 ± 0.24	0.98
**T2‐FLAIR volunteer**						
Overall image quality	3.58 ± 0.50	3.52 ± 0.51	3.48 ± 0.51	3.46 ± 0.50	3.52 ± 0.51	0.80
Gray–white matter delineation	3.50 ± 0.501	3.52 ± 0.51	3.48 ± 0.51	3.46 ± 0.50	3.52 ± 0.51	0.97
Overall diagnostic usefulness	3.50 ± 0.51	3.50 ± 0.51	3.50 ± 0.51	3.48 ± 0.51	3.48 ± 0.51	1.00
Contrast fat and rectus extraocular muscle	3.40 ± 0.50	3.52 ± 0.51	3.50 ± 0.51	3.50 ± 0.51	3.46 ± 0.50	0.73
Presence and severity of susceptibility artifacts	0.06 ± 0.24	0.04 ± 0.20	0.04 ± 0.20	0.04 ± 0.20	0.04 ± 0.20	0.99

Abbreviations: 3.0× = downsampling factor of 3.0, 3.5× = downsampling factor of 3.5, 4.0× = downsampling factor of 4.0, 4.5× = downsampling factor of 4.5, DL = deep learning–based reconstruction.

From the qualitative assessment results, it is observed that both the T1‐FLAIR and T2‐FLAIR deep learning–reconstructed images from different acceleration factors have statistically very similar scores as the fully sampled images of all the assessment metrics. It reflects that the reconstructed images from different acceleration factors have very similar image quality as the fully sampled images for diagnosis purposes with respect to radiologists, including the pathological regions for the clinical patients, as listed in Table 4. This result also matches with the diagnosis consistency study that both senior radiologists made the same diagnosis decision with respect to all the subjects (including both the healthy subjects and the clinical patients) of different acceleration factors. The accelerated reconstruction images produced by our proposed method demonstrated significantly superior subjective image quality scores compared to those generated by VARNET and the CycleGAN‐based network (Oh, Lee, and Ye 2021; Sriram et al. 2020), as shown in Table 5 and Figure 5.

**TABLE 5:** Qualitative assessment of different reconstruction methods under the 4.5× acceleration factor.

	Comparison Network 1	Comparison Network 2	Proposed method (DL 4.5×)	*p*
T1‐FLAIR	3.21 ± 0.14	3.36 ± 0.17	3.67 ± 0.24	< 0.01
T2‐FLAIR	3.22 ± 0.11	3.32 ± 0.14	3.66 ± 0.12	< 0.01

## 4 Discussion

The acceleration of MR scans has emerged as a pivotal focus within medical imaging due to its potential for significantly enhancing clinical workflows and patient satisfaction. This investigation leveraged a deep learning–based approach alongside multisequence information for expediting routine brain T1‐FLAIR/T2‐FLAIR sequence scans while systematically evaluating reconstructed images across varying acceleration factors. The primary advantages of deep learning are evident: It operates as a purely data‐driven method capable of autonomously determining optimal image recovery pathways for diverse acceleration factors and sequences without reliance on explicit prior assumptions inherent in traditional acceleration techniques (e.g., signal sparsity assumptions utilized in compressed sensing). Rigorous evaluations were conducted to appraise image quality resulting from deep learning–based reconstruction methods. Initial scrutiny focused on discerning whether these reconstructions led to differential diagnostic decisions compared with fully sampled radiologist‐made images. Furthermore, quantitative assessments employed SNR and CNR measurements across distinct anatomical regions for comparing deep learning–reconstructed versus fully sampled imagery. Subsequently, qualitative evaluation entailed comprehensive scoring of image quality across multiple dimensions by expert radiologists.

The proposed method offers several advantages over other state‐of‐the‐art deep learning–based methods. First, instead of solely accelerating a single sequence as many other fast reconstruction methods, the proposed approach incorporates an additional fully sampled sequence (T2WI in this study) that can be acquired relatively quickly to assist in reconstructing time‐consuming sequences (T1‐FLAIR and T2‐FLAIR in this study). The rich structural and anatomical details present in the fully sampled sequence significantly enhance the recovery of downsampled sequences, particularly at high acceleration factors. Second, the use of residual blocks in the reconstruction network ensures flexible information transfer between different network layers and reduces convergence challenges, especially for very deep networks. In addition, we employed a LSGAN training strategy, which demonstrates greater robustness compared to conventional cross‐entropy loss training strategies used by other GAN‐based deep learning–reconstruction methods (Mao et al. 2017).

Our model addressed challenging factors such as different clinical settings and patient demographic differences mainly in two ways. First, the patient cohort used to train the reconstruction model was carefully selected to cover as many clinical settings as possible, as both the healthy subjects and the patients who underwent brain scans were included between December 8, 2018 and March 11, 2019, where no specific disease was discarded or considered in a specific manner. The healthy subjects and the patients included in this study were also from different demographics admitted to the hospital, and no specific selection was performed. In this way, the model could be adapted to different clinical settings and patient demographics. Second, the reconstruction method was carefully designed to retain as many anatomical details as possible from the multiple sequences, especially from the fully sampled reference sequence, which can provide helpful information to the network to reconstruct the downsampled sequence. This design was not limited to specific scanner and can be generalized to different vendors and machines with different field strengths (e.g., 1.5 T), though in this study, data from the 3T scanner of United Imaging Healthcare were used

One important point to consider is how the method can be adapted to larger datasets, such as more anatomies and sequences. Techniques such as adjusting the size of the minibatch, data shuffling, and collaborative learning can be used to handle larger datasets (Anwar, Abrar, and Ullah 2023; Ullah et al. 2023a, 2023b, 2024, 2023c), and it will not fundamentally alter the time complexity of our approach. Also, to accommodate different image qualities, more data augmentation techniques could be applied to the original training images to increase the abundance of training materials and boost the generalizability of the model.

To integrate our method with diverse MRI hardware and software infrastructures, we also need to overcome several potential roadblocks, such as differences in MRI scanner manufacturer specifications, software interoperability, and data formatting issues. One potential strategy is to develop intelligent software wrappers that can intelligently adjust the model's parameters based on the individual MRI system's characteristics. It can significantly enhance our method's flexibility, enabling it to account for variations in image quality and noise levels inherent to different scanner setups. Furthermore, close collaboration with MRI scanner manufacturers could facilitate the inclusion of our method within their software suites, reducing the need for separate integration steps. Such collaborations might involve sharing specifications, joint testing, and developing dedicated plugin systems that marry the manufacturer's MRI software with our AI model.

This study has several limitations. First, the results were obtained using a 3T MRI scanner, and it remains uncertain whether the deep learning–based acceleration reconstruction method would yield similar performance on a 1.5 T MRI as demonstrated in this study. Second, while the investigation focused on the brain region using T1‐FLAIR and T2‐FLAIR sequences, further comprehensive assessments are required to explore the performance of deep learning–based acceleration reconstruction methods across other body regions and sequences. It should be noted that the computational complexity inherent in our AI‐based MRI reconstruction method could involve higher computational overhead compared to traditional techniques. To mitigate this, further optimization of our AI‐based algorithm and dedicated hardware acceleration, such as GPUs, was required to strike a balance between computational efficiency and scalability. In clinical applications, it is important to make sure critical diagnosis information was not lost in the reconstructed images, which is the basic principle in the trade‐offs between scanning speed and image quality. The proposed method might fail for patients with unique pathologies, which are not well‐represented in the training sets. The dataset used to train and evaluate the reconstruction model was carefully selected to cover as many clinical settings as possible, as both the healthy subjects and the patients who underwent brain scans were included between December 8, 2018 and March 11, 2019, where no specific disease was discarded or considered in a specific manner. The healthy subjects and the patients included in this study were also naturally admitted from different demographics to the hospital during that period, and no specific selection was performed. However, the generalizability and robustness of our method with respect to more geographic and demographic variations are yet to be verified.

Future research directions include extending the proposed method to other commonly scanned anatomies such as spines, knees, shoulders, and abdomen. Moreover, we can extend the proposed method to more MRI sequences, such as diffusion‐weighted imaging and dynamic contrast‐enhanced sequences. Other possible network structure designs, such as stable diffusion, could also be explored. It will also be important to investigate how to incorporate the Internet of Things (IoT) into AI‐driven MRI image reconstruction. It not only broadens the scope of enhanced imaging applications but also paves the way for a more interconnected healthcare system. IoT enables the seamless acquisition and transmission of patient data from MRI machines to AI algorithms in real‐time, facilitating faster reconstruction and even remote analysis and evaluations. This integration allows for intelligent scheduling for reconstruction resources, maintaining privacy and security, and driving innovation in medical imaging technology. Also, more sites may be covered and more patients with different diseases may be recruited to evaluate the proposed method. Finally, MRI machines, even when operating at the same field strength, can display differences due to variations in hardware components and vendor‐specific software implementations. This heterogeneity poses a challenge for AI models trained on data from a particular scanner, potentially leading to inconsistencies in findings across different imaging centers. To counter this, transfer learning and more generalized AI algorithms are crucial for maintaining diagnostic accuracy regardless of imaging hardware, which is also one of the further research directions.

## Author Contributions

**Zhanhao Mo**: conceptualization, data curation, formal analysis. **He Sui**: data curation, visualization, writing–original draft. **Zhongwen Lv**: resources, software, supervision. **Xiaoqian Huang**: conceptualization, investigation, methodology, project administration. **Guobin Li**: investigation, methodology, validation. **Dinggang Shen**: supervision, validation. **Lin Liu**: project administration, resources, supervision, visualization. **Shu Liao**: conceptualization, investigation, methodology, project administration, supervision, visualization, writing–review and editing.

## Ethics Statement

This retrospective study gained approval from the local institutional review board (IRB), with the IRB number of 20201112.

### Peer Review

The peer review history for this article is available at https://publons.com/publon/10.1002/brb3.70163.

## Data Availability

The data and code for this project are confidential but may be obtained from the corresponding author upon reasonable request.
